# Synthesis and Characterization of New Bivalent Agents as Melatonin- and Histamine H_3_-Ligands

**DOI:** 10.3390/ijms150916114

**Published:** 2014-09-12

**Authors:** Daniele Pala, Laura Scalvini, Alessio Lodola, Marco Mor, Lisa Flammini, Elisabetta Barocelli, Valeria Lucini, Francesco Scaglione, Silvia Bartolucci, Annalida Bedini, Silvia Rivara, Gilberto Spadoni

**Affiliations:** 1 Dipartimento di Farmacia, Università degli Studi di Parma, Parco Area delle Scienze 27/A, I-43124 Parma, Italy; E-Mails: daniele.pala@nemo.unipr.it (D.P.); laura.scalvini@studenti.unipr.it (L.S.); alessio.lodola@unipr.it (A.L.); marco.mor@unipr.it (M.M.); lisa.flammini@unipr.it (L.F.); elisabetta.barocelli@unipr.it (E.B.); 2 Dipartimento di Biotecnologie Mediche e Medicina Traslazionale, Università degli Studi di Milano, Via Vanvitelli 32, I-20129 Milano, Italy; E-Mails: valeria.lucini@unimi.it (V.L.); francesco.scaglione@unimi.it (F.S.); 3 Dipartimento di Scienze Biomolecolari, Università degli Studi di Urbino “Carlo Bo”, Piazza Rinascimento 6, I-61029 Urbino, Italy; E-Mails: silvia.bartolucci@uniurb.it (S.B.); annalida.bedini@uniurb.it (A.B.); gilberto.spadoni@uniurb.it (G.S.)

**Keywords:** melatonin receptor, MT_1_, MT_2_, H_3_ antagonists, bivalent ligands

## Abstract

Melatonin is an endogenous molecule involved in many pathophysiological processes. In addition to the control of circadian rhythms, its antioxidant and neuroprotective properties have been widely described. Thus far, different bivalent compounds composed by a melatonin molecule linked to another neuroprotective agent were synthesized and tested for their ability to block neurodegenerative processes *in vitro* and *in vivo*. To identify a novel class of potential neuroprotective compounds, we prepared a series of bivalent ligands, in which a prototypic melatonergic ligand is connected to an imidazole-based H_3_ receptor antagonist through a flexible linker. Four imidazolyl-alkyloxy-anilinoethylamide derivatives, characterized by linkers of different length, were synthesized and their binding affinity for human MT_1_, MT_2_ and H_3_ receptor subtypes was evaluated. Among the tested compounds, **14c** and **14d**, bearing a pentyl and a hexyl linker, respectively, were able to bind to all receptor subtypes at micromolar concentrations and represent the first bivalent melatonergic/histaminergic ligands reported so far. These preliminary results, based on binding affinity evaluation, pave the way for the future development of new dual-acting compounds targeting both melatonin and histamine receptors, which could represent promising therapeutic agents for the treatment of neurodegenerative pathologies.

## 1. Introduction

**Figure 1:**
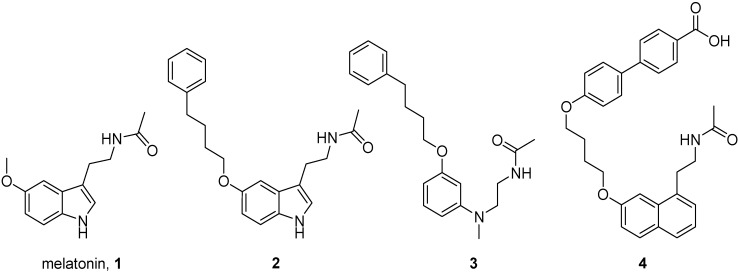
Structures of melatonin and of MT_1_-selective compounds bearing an aryl-alkyloxy chain.

Melatonin (**1**, Figure 1) is a tryptophan-derived hormone primarily secreted by the pineal gland according to a circadian rhythm, with peak concentrations at night. Most of the regulatory functions exerted by this hormone are mediated by two high-affinity G-protein-coupled receptors, named MT_1_ and MT_2_ [1], which are mainly expressed in the central nervous system (CNS) but are also present in different peripheral organs [2,3]. Nonetheless, melatonin has shown to bind also to several other cellular targets, including the MT_3_ binding site (quinone reductase 2), calmodulin, calreticulin, and tubulin [4], which could have a role in those phenotypic effects of melatonin that are independent of the activation of membrane-bound receptors. Additionally, melatonin effects could be also sustained by its metabolites, since it is rapidly transformed in the peripheral sites [5]. In addition to its well-established function in the regulation of the sleep-wake cycle and in the entrainment of circadian rhythms [6], melatonin is involved in a variety of other pathophysiological processes, including radical scavenging, attenuation of oxidative damage and neuroprotection [7,8,9,10]. Experimental evidence highlights the important role played by the activation of MT_1_ and MT_2_ receptors in sustaining its antioxidant and neuroprotective actions [11,12,13,14]. Even if the mechanism at the basis of this neuroprotective effect has not been completely elucidated, it is probably related to a multifactorial action exerted within the cell. For example, in motoneurons melatonin attenuated the production of reactive oxygen species, modulated Ca^2+^ levels and inhibited proapoptotic signaling [11,12]. In this context, not only melatonin but also synthetic compounds, like 2-iodomelatonin or other non-indole melatonergic ligands, have shown to efficiently produce antioxidant and cytoprotective effects and to block neurodegenerative processes both *in vitro* and *in vivo* [15,16]. Recently, different series of dual-acting compounds, constituted by melatonin linked to another known neuroprotective agent, have been reported as novel potential therapeutic agents for the treatment of neurodegenerative disorders. Indeed, hybrid melatonin-tacrine compounds showed potent anticholinesterase and antioxidant activity and melatonin-*N*,*N*-dibenzyl(*N*-methyl)amine hybrids also showed neuroprotective effects and were proposed as new potential therapeutic agents for neurodegenerative pathologies such as Parkinson’s and Alzheimer’s diseases. [17,18] Curcumin-melatonin hybrids showed antioxidant and neuroprotective actions as well, as demonstrated in *in vitro* studies on a cellular model of Alzheimer’s disease [19].

Histamine H_3_ receptors are mainly expressed in the CNS, with a predominantly presynaptic localization. They have been characterized as both autoreceptors and heteroreceptors, exerting a negative feedback mechanism on the synthesis and release of histamine and on the release of other neurotransmitters, such as acetylcholine, noradrenaline, dopamine, serotonin, *etc*. [20]. Histamine H_3_ receptor antagonists, which increase the release of such neurotransmitters, have been extensively investigated for the treatment of different CNS pathologies, such as narcolepsy, attention deficit hyperactivity disorder, obesity, and Alzheimer’s disease [21,22] and a number of potent and selective compounds are currently undergoing clinical trials. H_3_-antagonists have been also investigated for their neuroprotective potential and the improvement of cognitive disorders [23]. Several experimental studies evaluated the neuroprotective effects of different classes of H_3_ receptor antagonists *in vitro* and *in vivo* [24,25,26], highlighting their potential usefulness in the treatment of cognitive pathologies. Indeed, pretreatment with the H_3_ receptor antagonist ABT-239 was able to significantly attenuate kainic acid-mediated behavioral and excitotoxic effects [27]. Recently, a new class of compounds has been reported in which the pharmacophore for H_3_ receptor antagonists was combined with a 3-indolyl-alkyl portion. These compounds exhibited potent H_3_ receptor antagonist activity and free radical scavenging properties and were hypothesized to be superior agents for Alzheimer’s disease therapy by acting in a complementary manner [28].

Given the promising neuroprotective effects shown by melatonin receptor agonists and H_3_ receptor antagonists, we combined the pharmacophore elements of these classes into a single molecule, with the aim to retain the ability to bind both receptor subtypes. Structure-activity relationships (SARs) for melatonin receptor ligands showed that the introduction of an aryl-alkyloxy chain of suitable length in a position corresponding to that of the 5-methoxy group of melatonin is tolerated and leads to potent compounds selective for the MT_1_ subtype (e.g., compounds **2**–**4**, Figure 1) [29,30,31]. According to the results of docking studies on a homology model of the MT_1_ receptor, it has been hypothesized that the substituent conferring subtype selectivity can be accommodated within a lipophilic channel, exposed to the solvent at its cytosolic terminus, which is available in the MT_1_ receptor. In the MT_2_ receptor this channel is much more crowded, given the presence of bulkier amino acids hampering the accommodation of the aryl-alkyloxy chain in the same manner as in the MT_1_ receptor [31].

**Figure 2:**
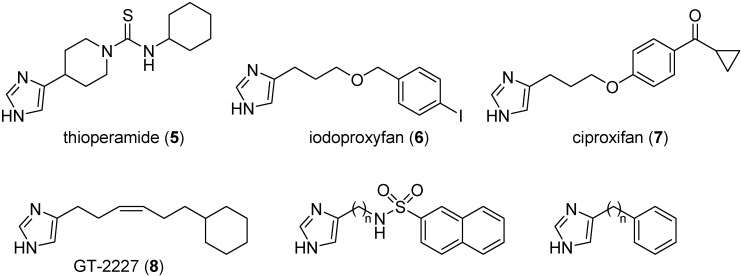
Structures of imidazole-based H_3_ antagonists.

The classical pharmacophore model for H_3_ receptor antagonists is composed by three main portions, *i.e.*, a basic group, a central lipophilic core usually connected through an alkyl spacer and a terminal group, which displays high chemical diversity as it could be a polar group, a lipophilic group or another basic center [32]. The first H_3_ receptor antagonists were imidazole derivatives, while the second generation of compounds carried a different basic group, usually a piperidine or a pyrrolidine. Several series of potent imidazole-based H_3_ antagonists lacking a second basic center have been reported [33]. A polar atom/group is often present in these compounds, such as a thiourea (compound **5**, Figure 2), a sulfonamide, or an oxygen atom (compounds **6** and **7**). Good antagonists were also obtained with the insertion of a lipophilic chain only (compound **8**), indicating that the presence of the imidazole ring is sufficient to preserve binding to the H_3_ receptor [34]. In addition, SARs for these non-basic imidazole-based H_3_ antagonists showed that the length of the alkyl linker can be significantly increased, sometimes reaching 9–10 methylene units, while maintaining significant H_3_ binding affinity [35,36,37,38].

The possibility of obtaining high H_3_ receptor binding affinity for compounds lacking a strongly basic center is an important point in light of the merging of the pharmacophore elements of H_3_-antagonists with those of melatonergic agonists. In fact, introduction of basic groups on the scaffold of melatonergic ligands invariably brought to a loss of binding affinity [39,40]. On the other hand, the tolerance shown by the H_3_ receptor for the length of the alkyl spacer supports the possibility of combining H_3_ receptor and melatonin receptor pharmacophore elements through a suitable alkyl spacer. In fact, good MT_1_ binding affinities could be obtained with aryl-alkyloxy substituents having alkyl chains of four methylene units or longer.

**Figure 3:**
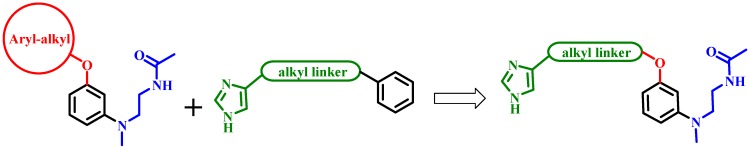
Schematic representation of the strategy followed in the design of dual melatonergic-H_3_ receptor ligands.

The melatonergic pharmacophore was provided by an anilinoethylamide fragment which already proved to bioisosterically reproduce the indol-3-ylethylamide portion of melatonin, affording compounds with similar binding affinity and intrinsic activity [41].Indeed, being the anilinoethylamide smaller than the indol-3-ylethylamide of melatonin, it could be better tolerated at the H_3_ receptor binding site. The anilinoethylamide fragment was decorated with an aryl-alkyloxy substituent containing the elements necessary for molecular recognition at the H_3_ receptor. The aryl-alkyloxy chain was a 4(5)-imidazolyl-alkyloxy one, seen in ciproxifan (**7**) and its analogs [42] (Figure 3).

**Figure 4:**
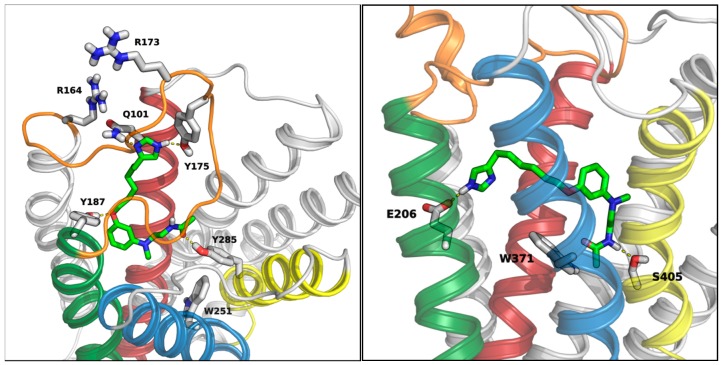
(**Left**) hypothetical binding conformation of compound **14d** (green carbons) within an MT_1_ receptor model (light gray carbons) [31]. Transmembrane helices 3, 5, 6 and 7 are colored red, green, blue and yellow, respectively, while extracellular loop 2 is depicted in orange; (**Right**) hypothetical binding conformation of **14d** within an H_3_ receptor model (light gray carbons) [45]. Transmembrane helices 3, 5, 6 and 7 are colored red, green, blue and yellow, respectively, while extracellular loop 2 is depicted in orange.

Docking studies suggested that these compounds could be accommodated within the MT_1_ receptor binding site. The 4(5)-imidazolyl-alkyl portion could occupy the lipophilic pocket delimited by transmembrane (TM) helices 3, 4, and 5, with the terminal imidazole ring positioned at the rim of the TM portion of the receptor, where it could undertake polar interactions with amino acids in helices or extracellular loops (Figure 4, left).

**Figure 5:**
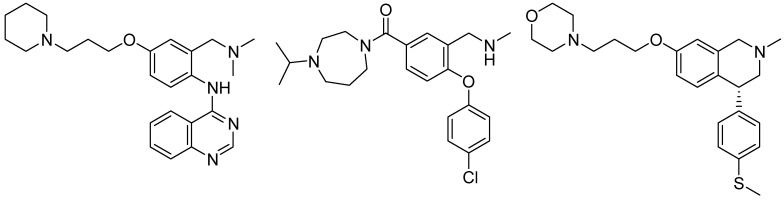
Dibasic H_3_ receptor antagonists carrying a lipophilic substituent.

The H_3_ receptor should be able to interact with these compounds by binding their imidazole ring through E206 on TM5, as supported by mutagenesis experiments on histamine and other imidazole-based ligands [43]. The anilinoethylamide fragment could be docked within a lipophilic cavity identified in H_3_ receptor models [44,45], mainly delimited by TM2, 6 and 7, and roughly perpendicular to the region where E206 is located (Figure 4, right). The existence of this lipophilic cavity is supported by the high binding affinity shown by H_3_ receptor antagonists with two basic centers and a lipophilic substituent that could extend over both binding site cavities (Figure 5) [45,46,47].

We report here the synthesis and evaluation of the binding affinity for histamine H_3_ and melatonin MT_1_ and MT_2_ receptors of four 3-(4(5)*-*imidazolyl-alkyloxy)-anilinoethylacetamides in which alkyl spacers of different length were introduced at position 3 of the aniline core, looking for an optimal linker to combine the H_3_ receptor- and the melatonin receptor-binding moieties.

## 2. Results and Discussion

### 2.1. Chemistry

**Scheme 1:**
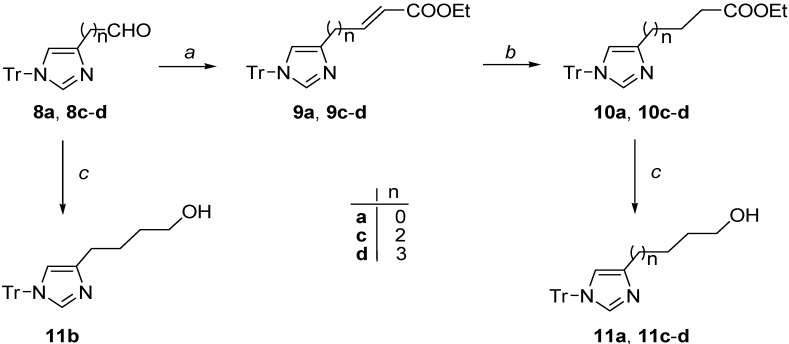
Synthesis of compounds **11a**–**d**.^a^

^a^ Reagents and conditions: (*a*) NaH, triethyl phosphonoacetate, THF, r.t.; (*b*) H_2_ (1 atm), 10% Pd/C, MeOH, 4 h, r.t.; (*c*) LiAlH_4_, THF, 4 h, reflux.

**Scheme 2:**
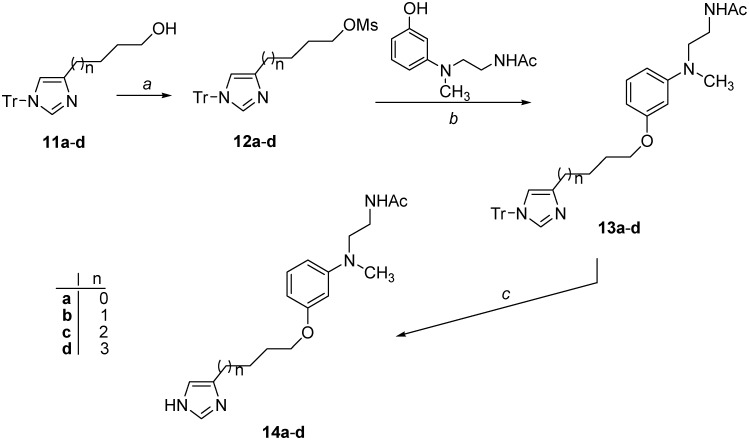
Synthesis of compounds **14a**–**d**.^a^

^a^ Reagents and conditions: (*a*) mesyl chloride, TEA, CH_2_Cl_2_, 1 h, 0 °C; (*b*) NaH, DMF, 16 h, r.t.; (*c*) 2N HCl, THF, 3 h, 70 °C.

The synthesis of the dual melatonergic/histaminergic ligands is described in Scheme 1 and Scheme 2.

The key starting alcohols **11a**, **11c**–**d** were prepared from the suitable aldehydes **8a**, **8c**–**d** following the previously reported three-step sequence (Wittig reaction, hydrogenation, LiAlH_4_ ester reduction) [48], whereas the alcohol **11b** could be obtained by direct reduction of the aldehyde **8d**[48] (Scheme 1).

The *N*-protected compounds **13a**–**d** were obtained by mesylation of the suitable (1*-*trityl-imidazol-4-yl)alkan-1-ol **11a**–**d**, followed by substitution of the intermediate methansulfonates **12a**–**d** with *N*-{2-[(3-hydroxyphenyl)methylamino]ethyl}acetamide [31] in the presence of NaH. Deprotection of the imidazole ring under acidic conditions finally yielded the target compounds **14a**–**d** (Scheme 2).

### 2.2. Binding Affinities of Compounds 14a–d for Melatonin MT_1_, MT_2_ and Histamine H_3_ Receptors

Binding affinities at human MT_1_, MT_2_ and H_3_ receptors of the newly synthesized *N*-(4(5)-imidazolyl-alkyloxy-anilinoethyl)acetamides **14a**–**d** were assessed as described in the Experimental Section and are reported in Table 1.

**Table 1:** Binding affinities (p*K*_i_) and intrinsic activities (*IA*_R_) measured for compounds **14a**–**d** at the human MT_1_, MT_2_ and H_3_ receptors. N.A. = Not active up to 100 μM. N.D. = Not determined.

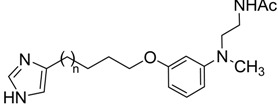						
Compound	*n*	hMT_1_		hMT_2_		hH_3_
		p*K*_i_	*IA*_R_	p*K*_i_	*IA*_R_	p*K*_i_
**1**		9.60 ± 0.18	1.00 ± 0.09	9.44 ± 0.12	1.00 ± 0.07	N.D.
**5**		N.D.	N.D.	N.D.	N.D.	7.28 ± 0.15
**14a**	0	N.A.		N.A.		5.91 ± 0.01
**14b**	1	N.A.		N.A.		N.D.
**14c**	2	6.09 ± 0.12	N.D.	6.28 ± 0.10	N.D.	6.28 ± 0.03
**14d**	3	6.79 ± 0.01	−0.26 ± 0.09	6.76 ± 0.06	−0.35 ± 0.19	6.22 ± 0.09

The bivalent melatonergic/histaminergic ligands differ for the length of the alkyl chain connecting the imidazole ring to the anilinoethylamide portion, spanning from three to six methylene units. Indeed, while for imidazole-based histamine H_3_ receptor ligands an ethyl or a propyl chain is usually preferred, for melatonergic ligands the optimal chain length is four methylene units or longer. Compound **14a** with the shortest spacer was totally inactive towards both MT_1_ and MT_2_ melatonin receptors, whereas it showed micromolar affinity for the H_3_ receptor. Elongation of the alkyl chain with a fourth methylene unit (**14b**) was not sufficient to achieve binding affinity towards melatonin receptors The first active bivalent ligand was obtained with a pentyl linker connecting the imidazole ring to the melatonergic fragment (**14c**). Indeed, although compound **14c** is significantly less potent than melatonin towards MT_1_ and MT_2_ receptors, it is able to bind to both melatonin and H_3_ receptor subtypes with micromolar affinity. A further elongation of the linker yielded the second active derivative **14d**, with improved binding affinity for melatonin receptors compared to **14c**. When evaluated in the GTPγS assay, **14c** behaved as an antagonist at both MT_1_ and MT_2_ receptors.

The low affinities of these compounds for melatonin receptors are likely to be ascribed to the presence of the imidazole ring. Indeed, compound **3** (Figure 1) displayed p*K*_i_ = 8.93 for MT_1_ receptors and, more generally, in the series of phenyl-alkyloxy-anilinoethylamides alkyl chains longer than four methylene units provided compounds with good binding affinities [31]. According to our docking hypothesis, the partial recovery of binding affinity observed with longer spacers could be due to the accommodation of the imidazole ring into a more peripheral, solvent-exposed region of the receptor. The limited binding affinity might be related to the presence, in this area, of some positively-charged amino acids belonging to extracellular loop 2 (e.g., R164 and R173 in the MT_1_ receptor) which could negatively interact with the imidazole ring (Figure 4). The presence of the imidazole ring is likely related to the antagonist behavior shown by **14d**. It remains to be evaluated if replacement of this ring with other heterocycles, tolerated by the H_3_ receptor, could restore the agonist activity.

Alkyl chain length seems not to significantly influence binding affinity at the H_3_ receptor. The longest derivatives **14c**–**d** are those with highest binding affinities, about 10 times lower than the reference imidazole-based H_3_ antagonist thioperamide (**5**). We may speculate that the anilinoethylamide portion undertakes some unfavorable interaction with the binding site which could contribute to the limited binding affinity observed for these compounds.

## 3. Experimental Section

### 3.1. General Experimental Procedures

^1^H NMR (200 MHz) and ^13^C NMR (50 MHz) spectra were recorded on a Bruker (Billerica, MA, USA) AVANCE 200 spectrometer, using CDCl_3_ as solvent. Chemical shifts (δ scale) are reported in parts per million (ppm) relative to the central peak of the solvent. Coupling constants (*J* values) are given in hertz (Hz). ESI-MS spectra were taken on a Waters (Milford, MA, USA) Micromass Zq instrument. Elemental analyses for C, H and N were performed on a Carlo Erba (Milan, Italy) analyzer, and the results are within 0.4% of the calculated values. UV-Vis spectra were recorded using a Beckman (Brea, CA, USA) DU640 spectrophotometer. Column chromatography purifications were performed under “flash” conditions using Merck 230–400 mesh silica gel. Analytical thin-layer chromatography (TLC) was carried out on Merck (Darmstadt, Germany) silica gel 60 F_254_ plates. Reagents were obtained from commercial suppliers and used without further purification. The aldehyde **8a** was commercially available; aldheydes **8c**–**d** [48] and *N*-{2-[(3-hydroxyphenyl)methylamino]ethyl}acetamide [31] were prepared as previously reported. The two radioligands 2-[^125^I]iodomelatonin (specific activity, 2000 Ci/mMol) and [^35^S]GTPγS ([^35^S]guanosine-5'-*O*-(3-thio-triphosphate); specific activity, 1000 Ci/mMol) were purchased from PerkinElmer (Waltham, MA, USA). [^3^H](*R*)-α-methylhistamine (specific activity, 47.0 Ci/mMol) was purchased from Amersham Bioscience (Amersham, UK).

### 3.2. Synthetic Procedures

#### 3.2.1. Synthesis of Unsaturated Esters **9a**, **9c**–**d**

General Procedure: A solution of triethyl phosphonoacetate (0.2 mL, 1 mMol) in THF (6 mL) was added to an ice-cooled suspension of NaH (80% dispersed in mineral oil, 0.030 g, 1 mMol) in THF (6 mL) and the resulting mixture was stirred at room temperature for 30 min. A solution of the opportune aldehyde **8a**, **8c**–**d** [48] (0.9 mMol) in THF (6 mL) was added dropwise and the mixture was stirred for 16 h at room temperature. The reaction mixture was then poured into iced water and extracted 3× with diethyl ether; the organic phases were combined, dried (Na_2_SO_4_) and concentrated to give a crude residue which was purified by flash chromatography (silica gel, EtOAc as eluent).

#### 3.2.2. (*E*)-Ethyl 3-(1-trityl-1*H*-imidazol-4-yl)acrylate (**9a**)

The chemical physical data are identical to those reported in literature [49].

#### 3.2.3. (*E*)-Ethyl 5-(1-trityl-1*H*-imidazol-4-yl)pent-2-enoate (**9c**)

ESI MS (*m*/*z*): 437 (M + 1)^+^; 243 (Ph_3_C^+^). The chemical physical data are identical to those reported in literature [48].

#### 3.2.4. (*E*-Ethyl 6-(1-trityl-1*H*-imidazol-4-yl)hex-2-enoate (**9d**)

Oil; 46% yield. ESI MS (*m*/*z*): 451 (M + 1)^+^; 243 (Ph_3_C^+^). ^1^H NMR (CDCl_3_): δ 1.29 (t, 3H *J* = 7.0), 1.76–1.88 (m, 2H), 2.17–2.24 (m, 2H), 2.54–2.61 (m, 2H), 4.18 (q, 2H *J* = 7.0), 5.78 (d, 1H, *J* = 16.0), 6.53 (s, 1H), 6.89–7.04 (dt, 1H, *J* = 7.0 and 16.0), 7.12–7.36 (m, 16H).

#### 3.2.5. Synthesis of Ester Derivatives **10a**, **10c**–**d**

General Procedure: A solution of the suitable ethyl ester **9a**, **9c**–**d** (1 mMol) in MeOH (5 mL) was hydrogenated under hydrogen atmosphere in the presence of 10% Pd/C (40 mg) for 4 h at room temperature. The catalyst was removed by filtration on Celite, and the filtrate was concentrated under reduced pressure to afford a crude residue which was purified by flash chromatography (silica gel, EtOAc as eluent).

#### 3.2.6. Ethyl 3-(1-trityl-1*H*-imidazol-4-yl)propanoate (**10a**)

White solid; 84% yield. ESI MS (*m*/*z*): 411 (M + 1)^+^, 243 (Ph_3_C^+^). ^1^H NMR (CDCl_3_): δ 1.24 (t, 3H, *J* = 7.0), 2.12 (t, 2H, *J* = 7.5), 2.64 (t, 2H, *J* = 7.5), 4.15 (q, 2H, *J* = 7.0), 6.63 (s, 1H), 7.14–7.39 (m, 16H).

#### 3.2.7. Ethyl 5-(1-trityl-1*H*-imidazol-4-yl)pentanoate (**10c**)

ESI MS (*m*/*z*): 439 (M + 1)^+^; 243 (Ph_3_C^+^). The chemical physical data are identical to those reported in literature [48].

#### 3.2.8. Ethyl 6-(1-trityl-1*H*-imidazol-4-yl)hexanoate (**10d**)

Oil; 86% yield. ESI MS (*m*/*z*): 453 (M + 1)^+^, 243 (Ph_3_C^+^). ^1^H NMR (CDCl_3_): δ 1.25 (t, 3H *J* = 7.0), 1.25–1.42 (m, 2H), 1.57–1.72 (m, 4H), 2.25–2.32 (m, 2H), 2.51–2.59 (m, 2H), 4.12 (q, 2H *J* = 7.0), 6.53 (s, 1H), 7.12–7.38 (m, 16H).

#### 3.2.9. Synthesis of Alcohol Derivatives **11a**, **11c**–**d**

General Procedure: A solution of appropriate ester **10a**, **10c**–**d** (1 mMol) in dry THF (4 mL) was added dropwise to a suspension of LiAlH_4_ (0.076 g, 2 mMol) in dry THF (4 mL) and the resulting mixture was refluxed for 4 h. After cooling to 0 °C the reaction mixture was quenched by slow addition of a saturated aqueous solution of NaHCO_3_ and EtOAc. The mixture was filtered on Celite, the filtrate was concentrated under reduced pressure to afford a residue which was taken up in CH_2_Cl_2_ and washed with a saturated aqueous solution of NaHCO_3_. The organic phase was dried (Na_2_SO_4_) and concentrated *in vacuo* to give a crude product which was purified by flash chromatography (silica gel, EtOAc to EtOAc/MeOH 97/3 as eluent).

#### 3.2.10. 3-(1-Trityl-1*H*-imidazol-4-yl)propan-1-ol (**11a**)

ESI MS (*m*/*z*): 369 (M + 1)^+^; 243 (Ph_3_C^+^). The chemical physical data are identical to those reported in literature [48].

#### 3.2.11. 4-(1-Trityl-1*H*-imidazol-4-yl)butan-1-ol (**11b**)

The product was obtained by direct reduction of the aldehyde **8d** as previously reported and the chemical physical data are identical to those reported in literature [48]. ESI MS (*m*/*z*): 383 (M + 1)^+^; 243 (Ph_3_C^+^).

#### 3.2.12. 5-(1-Trityl-1*H*-imidazol-4-yl)pentan-1-ol (**11c**)

ESI MS (*m*/*z*): 397 (M + 1)^+^; 243 (Ph_3_C^+^). The chemical physical data are identical to those reported in literature [48].

#### 3.2.13. 6-(1-Trityl-1*H*-imidazol-4-yl)hexan-1-ol (**11d**)

Oil; 94% yield. ESI MS (*m*/*z*): 411 (M + 1)^+^, 243 (Ph_3_C^+^). ^1^H NMR (CDCl_3_): δ 1.23–1.39 (m, 4H), 1.48–1.67 (m, 4H), 2.52–2.60 (m, 2H), 3.59–3.65 (m, 2H), 6.52 (s, 1H), 7.09–7.40 (m, 16H).

#### 3.2.14. Synthesis of Mesyl Derivatives **12a**–**d**

General Procedure: Methanesulfonyl chloride (0.25 g, 2.2 mMol) was added to an ice-cooled solution of the opportune alcohol **11a**–**d** (1.8 mMol) in dry CH_2_Cl_2_ (8 mL) and TEA (0.24 g, 2.4 mMol). The reaction mixture was stirred under nitrogen atmosphere at 0 °C for 1 h (for **10a** further 3 h at room temperature), quenched with water and washed once with an aqueous solution of 5% NaHCO_3_. The organic phase was dried over Na_2_SO_4_ and the solvent removed by distillation to afford the crude mesylate (**12a**–**d**), which was used in the next step without any further purification.

#### 3.2.15. 3-(1-Trityl-1*H*-imidazol-4-yl)propyl methansulfonate (**12a**)

Oil; 92% yield. ESI MS (*m*/*z*): 447 (M + 1)^+^, 243 (Ph_3_C^+^). ^1^H NMR (CDCl_3_): δ 2.11 (quint, 2H), 2.65–2.77 (m, 2H), 2.97 (s, 3H), 4.26 (t, 2H), 6.59 (s, 1H), 7.12–7.44 (m, 16H).

#### 3.2.16. 4-(1-Trityl-1*H*-imidazol-4-yl)butyl methansulfonate (**12b**)

Oil; 95% yield. ESI MS (*m*/*z*): 461 (M + 1)^+^, 243 (Ph_3_C^+^).

#### 3.2.17. 5-(1-Trityl-1*H*-imidazol-4-yl)pentyl methansulfonate (**12c**)

Oil; 87% yield. ESI MS (*m*/*z*): 475 (M + 1)^+^, 243 (Ph_3_C^+^).

#### 3.2.18. 6-(1-Trityl-1*H*-imidazol-4-yl)hexyl methansulfonate (**12d**)

Oil; 88% yield. ESI MS (*m*/*z*): 489 (M + 1)^+^, 243 (Ph_3_C^+^).

#### 3.2.19. Synthesis of Derivatives **13a**–**d**

General Procedure: NaH (80% in mineral oil, 0.036 g, 1.2 mMol) was added to a solution of *N*-{2-[(3-hydroxyphenyl)methylamino]ethyl}acetamide [31] (0.21 g, 1 mMol) in dry DMF (2.2 mL) under nitrogen atmosphere. After stirring for 30 min at room temperature, a solution of the suitable mesyl derivative **12a**–**d** (1 mMol) in dry DMF (1 mL) was added to the reaction mixture and stirring continued for 16 h. The reaction mixture was poured into water and extracted 3× with EtOAc. The organic phases were combined, washed once with brine, dried (Na_2_SO_4_) and concentrated to give a crude residue, which was purified by flash chromatography (silica gel, EtOAc as eluent).

#### 3.2.20. *N*-[2-(Methyl{3-[3-(1-trityl-1*H*-imidazol-4-yl)propoxy]phenyl}amino)ethyl]acetamide (**13a**)

Oil; 67% yield. ESI MS (*m*/*z*): 559 (M + 1)^+^, 243 (Ph_3_C^+^). ^1^H NMR (CDCl_3_): δ 1.92 (s, 3H), 2.06-2.18 (m, 2H), 2.74 (t, 2H, *J* = 7.5), 2.93 (s, 3H), 3.45 (m, 4H), 3.98 (t, 2H, *J* = 6.5), 5.86 (brs, 1H), 6.25 (dd, 1H, *J* = 2.0 and 8.0), 6.27 (m, 1H), 6.36 (dd, 1H, *J* = 2.0 and 8.0), 6.58 (s, 1H), 7.08–7.38 (m, 17H).

#### 3.2.21. *N*-[2-(Methyl{3-[4-(1-trityl-1*H*-imidazol-4-yl)butoxy]phenyl}amino)ethyl]acetamide (**13b**)

Oil; 40% yield. ESI MS (*m*/*z*): 573 (M + 1)^+^, 243 (Ph_3_C^+^). ^1^H NMR (CDCl_3_): δ 1.67–1.78 (m, 4H), 1.91 (s, 3H), 2.75 (t, 2H), 2.94 (s, 3H), 3.48 (m, 4H), 4.00 (t, 2H), 5.82 (brs, 1H), 6.26 (dd, 1H, *J* = 2.0 and 8.0), 6.29 (m, 1H), 6.38 (dd, 1H, *J* = 2.0 and 8.0), 6.59 (s, 1H), 7.06–7.41 (m, 17H). ^13^C NMR (CDCl_3_): δ 170.4, 160.3, 150.8, 142.3, 141.1, 138.0, 129.9, 129.7, 128.0, 117.9, 105.4, 102.4, 99.7, 75.3, 67.6, 51.7, 38.4, 37.2, 29.7, 28.9, 27.9 25.8, 23.2.

#### 3.2.22. *N*-[2-(Methyl{3-[5-(1-trityl-1*H*-imidazol-4-yl)pentyloxy]phenyl}amino)ethyl]acetamide (**13c**)

Oil; 71% yield. ESI MS (*m*/*z*): 587 (M + 1)^+^, 243 (Ph_3_C^+^). ^1^H NMR (CDCl_3_): δ 1.44–1.55 (m, 2H), 1.62–1.82 (m, 4H), 1.91 (s, 3H), 2.57 (t, 2H *J* = 7.5), 2.92 (s, 3H), 3.41–3.44 (m, 4H), 3.93 (t, 2H *J* = 6.5), 5.96 (brs, 1H), 6.25 (dd, 1H, *J* = 2.0 and 8.0), 6.27 (m, 1H), 6.36 (dd, 1H, *J* = 2.0 and 8.0), 6.54 (s, 1H), 7.10–7.36 (m, 17H). ^13^NMR (CDCl_3_): δ 170.4, 160.4, 150.8, 142.5, 141.7, 138.2, 129.9, 129.8, 128.0, 117.8, 105.4, 102.3, 99.7, 75.1, 67.8, 51.7, 38.4, 37.3, 29.5, 29.2, 29.1, 28.3, 25.8, 23.2.

#### 3.2.23. *N*-[2-(Methyl{3-[6-(1-trityl-1*H*-imidazol-4-yl)hexyloxy]phenyl}amino)ethyl]acetamide (**13d**)

Oil; 85% yield. ESI MS (*m*/*z*): 601 (M + 1)^+^, 243 (Ph_3_C^+^). ^1^H NMR (CDCl_3_): δ 1.26–1.52 (m, 4H), 1.53–1.75 (m, 4H), 1.95 (s, 3H), 2.60 (t, 2H *J* = 7.5), 2.94 (s, 3H), 3.45–3.47 (m, 4H), 3.94 (t, 2H *J* = 6.5), 5.75 (brs, 1H), 6.27 (dd, 1H, *J* = 2.0 and 8.0), 6.29 (m, 1H), 6.37 (dd, 1H, *J* = 2.0 and 8.0), 6.57 (s, 1H), 7.13–7.35 (m, 17H).

#### 3.2.24. Synthesis of Target Derivatives **14a**–**d**

General Procedure: A solution of the suitable trityl derivative **13a**–**d** (0.5 mMol) and 2 N HCl (2 mL) in THF (1 mL) was heated at 70 °C for 3 h. The reaction mixture was washed with diethyl ether, the aqueous phase basified with 2 N Na_2_CO_3_ and extracted 3× with diethyl ether. The organic phases were combined, dried (Na_2_SO_4_) and concentrated to give a residue that was purified by filtration through a silica gel plug (EtOAc-MeOH 85:15 as eluent).

#### 3.2.25. *N*-[2-({3-[3-(1*H*-Imidazol-4-yl)propoxy]phenyl}methylamino)ethyl]acetamide (**14a**)

Oil; 57% yield. ESI MS (*m*/*z*): 317 (M + 1)^+^. ^1^H NMR (CDCl_3_): δ 1.96 (s, 3H), 2.06–2.15 (m, 2H), 2.83 (t, 1H, *J* = 7.0), 2.94 (s, 3H), 3.45 (m, 4H), 4.03 (t, 1H, *J* = 6.5), 6.10 (brs, 1H), 6.26–6.37 (m, 3H), 6.83 (s, 1H), 7.13 (dd, 1H, *J*_1_* = J*_2_ = 8.0), 7.60 (s, 1H). ^13^C NMR (CDCl_3_): δ 170.9, 160.2, 150.7, 135.6, 134.5, 130.0, 118.3, 105.3, 102.6, 99.3, 67.0, 51.6, 38.4, 37.1, 29.7, 29.3, 23.2. UV-Vis (MeOH) λ = 253 (12,600), 293 (4300) nm (ε). Anal. calcd for C_17_H_24_N_4_O_2_: C 64.53, H 7.65, N 17.71, found: C 64.29, H 7.70, N 17.39.

#### 3.2.26. *N*-[2-({3-[4-(1*H*-Imidazol-4-yl)butoxy]phenyl}methylamino)ethyl]acetamide (**14b**)

Oil; 66% yield. ESI MS (*m*/*z*): 331 (M + 1)^+^. ^1^H NMR (CDCl_3_): δ 1.86–1.90 (m, 2H), 1.99 (s, 3H), 2.75–2.82 (m, 4H), 2.95 (s, 3H), 3.46 (m, 4H), 4.02 (t, 2H *J* = 6.5), 6.24 (brs, 1H), 6.26–6.41 (m, 3H), 6.97 (s, 1H), 7.13 (dd, 1H, *J*_1_ *= J*_2_ = 8.0), 7.93 (s, 1H). ^13^C NMR (CDCl_3_): δ 170.9, 160.2, 150.7, 136.3, 133.6, 130.0, 117.5, 105.4, 102.4, 99.7, 67.5, 51.7, 38.4, 37.1, 28.5, 25.9, 25.7, 23.2. Anal. calcd for C_18_H_26_N_4_O_2_: C 65.43, H 7.93, N 16.96, found: C 65.18, H 8.01, N 16.60.

#### 3.2.27. *N*-[2-({3-[5-(1*H*-Imidazol-4-yl)pentyloxy]phenyl}methylamino)ethyl]acetamide (**14c**)

Oil; 92% yield. ESI MS (*m*/*z*): 345 (M + 1)^+^. ^1^H NMR (CDCl_3_): δ 1.43–1.56 (m, 2H), 1.60–1.84 (m, 4H), 1.95 (s, 3H), 2.67 (t, 2H *J* = 7.0), 2.94 (s, 3H), 3.45–3.47 (m, 4H), 3.96 (t, 2H *J* = 6.5), 6.05 (brs, 1H), 6.25–6.37 (m, 3H), 6.81 (s, 1H), 7.13 (dd, 1H, *J*_1_ *= J*_2_ = 8.0), 7.61 (s, 1H). ^13^C NMR (CDCl_3_): δ 170.9, 160.2, 150.7, 136.1, 134.1, 129.9, 118.0, 105.3, 102.2, 99.5, 67.5, 51.6, 38.4, 37.2, 28.9, 28.9, 26.2, 25.6, 23.2. UV-Vis (MeOH) λ = 253 (13,000), 293 (4100) nm (ε). Anal. calcd for C_19_H_28_N_4_O_2_: C 66.25, H 8.19, N 16.27, found: C 66.03, H 8.16, N 15.88.

#### 3.2.28. *N*-[2-({3-[6-(1*H*-Imidazol-4-yl)hexyloxy]phenyl}methylamino)ethyl]acetamide (14d)

Oil; 84% yield. ESI MS (*m*/*z*): 359 (M + 1)^+^. ^1^H NMR (CDCl_3_): δ 1.41–1.47 (m, 4H), 1.61–1.80 (m, 4H), 1.94 (s, 3H), 2.64 (t, 2H *J* = 7.0), 2.94 (s, 3H), 3.45–3.47 (m, 4H), 3.95 (t, 2H *J* = 6.5), 5.88 (brs, 1H), 6.27–6.39 (m, 3H), 6.78 (s, 1H), 7.13 (dd, 1H, *J*_1_* = J*_2_ = 8.0), 7.58 (s, 1H). ^13^C NMR (CDCl_3_): 170.7, 160.3, 150.8, 136.3, 134.1, 129.9, 117.8, 105.3, 102.2, 99.6, 67.6, 51.7, 38.4, 37.3, 29.1, 29.1, 28.7, 26.2, 25.7, 23.2. UV-Vis (MeOH) λ = 252 (13,200), 294 (4100) nm (ε). Anal. calcd for C_20_H_30_N_4_O_2_: C 67.01, H 8.44, N 15.63, found: C 66.70, H 8.42, N 15.33.

### 3.3. Human Histamine H_3_ Receptor Binding Assay

Homogenates of SK–N–MC cells expressing human histamine H_3_ receptors (hH_3_Rs) were used to determine affinity values of the new compounds in radioligand displacement studies according to Lovenberg *et al*.’s method [50]. SK–N–MC cells in confluent culture plates were harvested using trypsin digestion and then centrifuged for 5 min at 1100 rpm. Pellets, either fresh or stored at −80 °C until the moment of use, were mechanically homogenized with Potter-Elvhejem in 20 mM Tris-HCl/0.5 mM EDTA. Supernatants from a 2000 rpm spin (10 min) were collected and re-centrifuged at 10,000 rpm for 30 min. Pellets were re-homogenized in 50 mM Tris-HCl/5 mM EDTA (pH 7.4). Membranes were incubated for 60 min at room temperature with 0.5 nM [^3^H](*R*)-α-methylhistamine in 50 mM Tris-HCl/5 mM EDTA (pH 7.4) with or without competing ligands. Each concentration was tested in triplicate. Incubation was terminated by rapid filtration over Millipore (Darmstadt, Germany) AAWPO2500 filters followed by two washes with ice-cold buffer (50 mM Tris-HCl/5 mM EDTA). Filters retained radioactivity was determined by liquid scintillation counting (Ultima Gold XR Perkin Elmer (Waltham, MA, USA) scintillation liquid, Tri-Carb 2810 TR Perkin Elmer (Waltham, MA, USA) Liquid Scintillation Analyzer). Non-specific binding was defined with 10 μM histamine as competing ligand. p*IC*_50_ and Hill coefficient values were estimated from the displacement curves of the tested compounds (10 nM–100 μM) *versu*s [^3^H](*R*)-α-methylhistamine, using Prism (San Diego, CA, USA) GraphPad 2005, and converted into p*K*_i_ values according to the Cheng–Prusoff equation [51]. Data are expressed as mean ± SEM of three independent experiments.

### 3.4. Human Melatonin Receptors Binding Assay

Binding affinities were determined using 2-[^125^I]iodomelatonin as the labeled ligand in competition experiments with cloned human MT_1_ and MT_2_ receptors expressed in NIH3T3 rat fibroblast cells. The characterization of NIH3T3 MT_1_ and MT_2_ cells was already described in detail [52,53]. Membranes were incubated for 90 min at 37 °C in binding buffer (50 mM Tris/HCl, pH 7.4). The final membrane concentration was 5–10 μg of protein per tube. The membrane protein level was determined in accordance with a previously reported method [54]. 2-[^125^I]Iodomelatonin (100 pM) and different concentrations of the tested compounds were incubated with the receptor preparation for 90 min at 37 °C. Nonspecific binding was assessed with 10 μM melatonin; *IC*_50_ values were determined by nonlinear fitting strategies with Prism (GraphPad SoftWare Inc., San Diego, CA, USA). The p*K*_i_ values were calculated from the IC_50_ values according to the Cheng–Prusoff equation [51]. The p*K*_i_ values are the mean of at least three independent determinations performed in duplicate.

To determine the functional activity of compound **14d** at MT_1_ and MT_2_ receptor subtypes, [^35^S]GTPγS binding assays in NIH3T3 cells expressing human-cloned MT_1_ or MT_2_ receptors were performed. The amount of bound [^35^S]GTPγS is proportional to the level of the analog-induced G protein activation and is related to the intrinsic activity of the compound under study. The detailed description and validation of this method were reported elsewhere [52,55]. Membranes (15–25 μg of protein, final incubation volume 100 μL) were incubated at 30 °C for 30 min in the presence and absence **14d**, in assay buffer consisting of [^35^S]GTPγS (0.3–0.5 nM), GDP (50 μM), NaCl (100 mM), and MgCl2 (3 mM). Nonspecific binding was defined using GTPγS (10 μM). In cell lines expressing human MT_1_ or MT_2_ receptors, melatonin produced concentration-dependent stimulation of basal [^35^S]GTPγS binding with a maximal stimulation above basal levels of 370% and 250% in MT_1_ and MT_2_ receptors, respectively. Basal stimulation is the amount of [^35^S]GTPγS specifically bound in the absence of compounds and was taken as 100%. The maximal G protein activation was measured in each experiment using melatonin (100 nM). Compounds were added at three different concentrations (one concentration equivalent to 100 nM melatonin, a second one, 10-fold smaller, and a third one, 10-fold larger), and the percent stimulation above basal was determined. The equivalent concentration was estimated on the basis of the ratio of the affinity of the test compound to that of melatonin. It was assumed that, at the equivalent concentration, the test compound occupies the same number of receptors as 100 nM melatonin. All of the measurements were performed in triplicate. The relative intrinsic activity (IA_R_) values were obtained by dividing the maximum ligand induced stimulation of [^35^S]GTPγS binding by that of melatonin, as measured in the same experiment.

### 3.5. Docking Studies

All docking simulations were performed with Glide 5.7 [56]; Maestro 9.2 [57] was applied to prepare ligand structures and to refine protein-ligand complexes.

#### 3.5.1. MT_1_ Receptor

A previously-reported MT_1_ receptor model [31] was taken as starting point for induced-fit docking (IFD) of compounds **14a**–**d**. An initial softened-potential docking run was performed applying van der Waals radii scaling of 0.7 and 0.5 on protein and ligand non-polar atoms, respectively. Amino acids hampering the accommodation of the imidazole ring of **14a**–**d**, *i.e.*, Q101 and Q169, were temporarily mutated to alanines. Energy grids generated for the initial softened-potential docking were centered in the putative binding site of the receptor, setting enclosing and bounding boxes to default dimensions. During flexible docking runs**,** two hydrogen bond constraints were applied between Y187 hydroxyl group and the phenolic oxygen of the ligand, and between Y285 hydroxyl group and the amide oxygen of the ligand, to reproduce the main polar interactions previously proposed for melatonergic ligands. Ligand docking was performed in standard precision (SP) mode, collecting fifty poses. The resulting ligand–receptor complexes were then submitted to a protein structure refinement stage; once amino acid side chains that had previously been removed were re-introduced, residues within a shell of 5 Å around any ligand pose were refined by a side chain conformational search, followed by energy minimization of the residues and the ligand molecule. In the final docking stage, each ligand structure obtained at the end of the protein structure refinement was energetically optimized (refined) in the field of the receptor and subsequently scored using default Glide settings. The final ligand-protein complexes were ranked according to their IFD score, a composite score that accounts for ligand-receptor interaction energy, receptor strain and solvation terms. The best-ranked MT_1_-**14a**–**d** complexes were minimized applying the OPLS2005 force field [58] to a convergence threshold of 0.05 kJ·mol^−1^·Å^−1^. During this minimization procedure, the ligand and residues within 8 Å from the ligand were free to move, while all other atoms were retained fixed.

#### 3.5.2. H_3_ Receptor

A previously-reported model of the histamine H_3_ receptor [45] was used as reference structure for docking studies of compounds **14a**–**d**. Glide grids were centered in the putative binding site of the receptor, located between D114 and E206, setting the dimension of enclosing and bounding boxes to default values. Compounds **14a**–**d** were flexibly docked within the H_3_ receptor binding cavity in SP mode, applying a van der Waals radii scaling of 0.7 on ligand non-polar atoms. During docking runs, a hydrogen bond constraint was applied between E206 and the imidazole ring of the ligands. Fifty poses were collected for each ligand and ranked according to their Emodel value. The best-scored ligand conformations were merged into the H_3_ receptor model and the resulting complexes were minimized applying the OPLS2005 force field to an energy gradient of 0.05 kJ·mol^−1^ Å^−1^. During this minimization phase, the docked compound and all residues within 8 Å from the ligand were free to move, whereas all other atoms were retained fixed.

## 4. Conclusions

We reported the synthesis and binding affinity evaluation of a series of bivalent melatonergic/histaminergic ligands characterized by an imidazolyl-alkyloxy-anilinoethylamide structure. In this series, four derivatives with alkyl linkers of different length were tested for their ability to bind human MT_1_, MT_2_ and H_3_ receptor subtypes. Among the tested compounds, **14c** and **14d**, bearing a pentyl and a hexyl linker between the imidazole ring and the aniline core, respectively, were able to bind to all receptor subtypes at micromolar concentrations. Although these two compounds might possess sub-optimal binding affinities, they are the first bivalent melatonergic/histaminergic ligands reported so far and might represent promising starting points for the development of potent dual-acting agents, potentially useful for the treatment of cognitive disorders.
